# Experimental and Numerical Investigation of Bond-Slip Behavior of High-Strength Reinforced Concrete at Service Load

**DOI:** 10.3390/ma15010293

**Published:** 2021-12-31

**Authors:** Alinda Dey, Domas Valiukas, Ronaldas Jakubovskis, Aleksandr Sokolov, Gintaris Kaklauskas

**Affiliations:** 1 Department of Reinforced Concrete Structures and Geotechnics, Vilnius Tech University (VGTU), Saulatekio al. 11, 10221 Vilnius, Lithuania; domas.valiukas@vilniustech.lt (D.V.); ronaldas.jakubovskis@vilniustech.lt (R.J.); gintaris.kaklauskas@vilniustech.lt (G.K.); 2 Laboratory of Innovative Building Structures, Vilnius Tech University (VGTU), Saulatekio al. 11, 10221 Vilnius, Lithuania; aleksandr.sokolov@vilniustech.lt

**Keywords:** reinforced concrete, double pull-out test, bond-slip, finite element modelling

## Abstract

A bond mechanism at the reinforcement-concrete interface is one of the key sources of the comprehensive functioning of reinforced concrete (RC) structures. In order to apprehend the bond mechanism, the study on bond stress and slip relation (henceforth referred as bond-slip) is necessary. On this subject, experimental and numerical investigations were performed on short RC tensile specimens. A double pull-out test with pre-installed electrical strain gauge sensors inside the modified embedded rebar was performed in the experimental part. Numerically, a three dimensional rib scale model was designed and finite element analysis was performed. The compatibility and reliability of the numerical model was verified by comparing its strain result with an experimentally obtained one. Afterwards, based on stress transfer approach, the bond-slip relations were calculated from the extracted strain results. The maximum disparity between experimental and numerical investigation was found as 19.5% in case of strain data and 7% for the bond-slip relation at the highest load level (110 kN). Moreover, the bond-slip curves at different load levels were compared with the bond-slip model established in CEB-fib Model Code 2010 (MC2010). Overall, in the present study, strain monitoring through the experimental tool and finite element modelling have accomplished a broader picture of the bond mechanism at the reinforcement-concrete interface through their bond-slip relationship.

## 1. Introduction

Reinforced concrete (RC) is a composite material designed from different types of concrete and reinforcing bars. The reinforcing bars are strategically placed in the tensile areas of RC structures, resisting tension forces after cracking of concrete. Consequently, the interaction between concrete and reinforcement bars, often referred to as bond, is essential in developing the required performance of the structure.

Modern non-destructive tests can be a possible option for the assessment of the conditions of concrete structures [1]. However, in case of RC structures, researchers relied on a number of different experimental techniques to study bond action ever since its invention. The most common techniques are based on the measured force-displacement relationships of the reinforcing bar pulled from the concrete [2]. In such tests (pull-out, push-in, beam, or beam end set-ups), the average bond stress distribution in the anchorage length as a function of relative displacement (or slip) may be obtained [3]. The measured bond-slip response from these tests served as a basis for several well-known bond-slip relationships [4,5,6]. Nevertheless, non-uniform bond stress distribution, compressive stress fields, structural deformation of the specimen, and a different slip at the loaded and free ends may misrepresent the “local” bond-slip phenomenon [7]. Moreover, the force-displacement experimental set up represents the average bond stress within the anchorage length and bond deterioration effect at the loaded end of reinforcement cannot be directly evaluated [8]. As the anchorage length in the pull-out test set ups is kept at the minimum [2] (generally less that 5D, where D is the bar diameter), the bond deterioration may significantly distort the “local“ bond-slip relationships.

As an alternative to traditional force displacement based experimental arrangements, a direct strain measurement of reinforcement strain inside the concrete element may be applied to study the bond-slip behavior [9,10]. In fact, bond stress and slip along a bar can be derived from the reinforcement strain profile; bond stress is proportional to the reinforcement strain gradient, whereas slip may be calculated through the integration of the reinforcement strain curve [6,11]. A double pull-out experimental set up (or tensile RC element) may be considered as a good balance between simplicity and realistic representation of the reinforcement and concrete interaction in the tensile zones of the RC structure [12].

Experimentally measured reinforcement strain distribution in a tensile RC element gives valuable data on the force transfer mechanism from reinforcement to concrete, as well as the development of slip along the element. However, the diffusion of tensile stresses in the concrete, micro-cracking, and internal damage of concrete can hardly be measured experimentally [13]. In this respect, numerical finite element (FE) models can be used for the deeper understanding of bond action, particularly the contribution of concrete to the force transfer mechanism. The FE models may be applied for different scales of bond analysis: rib, bar, and member scales [14]. Among these, rib-scale FE models were commonly applied for problems desiring high accuracies, such as bond damage in shear, concrete fracture at the interface with reinforcement, or modelling complex formation of secondary and longitudinal cracks [15,16]. Recently, it was shown that a refined rib-scale FE model can also be employed to study the reinforcement strain profile as well as the internal cracking of concrete in the tensile RC prisms; these may be accurately represented using a refined rib-scale FE models [17]. Such models allow an in-depth analysis of not only the distribution of bond stresses and slip, but also the deterioration of concrete at the loaded end of reinforcement [8].

Although several well-documented experimental campaigns have been carried out measuring the strain distribution in the tensile RC prisms [6,10,18,19], there is limited research on a bond action in high strength concrete elements. The differences in bond behavior in RC elements produced from high-strength concrete may possibly occur at the advanced loading stages, with intense propagation of internal cracking. Consequently, fracture energy may be one of the key parameters controlling bond action at high loadings. This paper presents an experimental and numerical investigation of reinforcement and concrete interaction in a tensile high-strength RC element, equipped with internally distributed electrical strain gauges. Furthermore, a detailed rib-scale numerical model was developed for the comprehensive analysis of bond action. The obtained results revealed that the bond transfer mechanism in such element may be considerably different from the commonly applied design bond-slip relationships.

## 2. Laboratory Experiment

The experiment was performed by preparing a RC prism of cross-section 200 mm × 200 mm and the length of 390 mm with 1–20 mm ϕ reinforcement placed concentrically along its longitudinal axis. These dimensions were chosen considering one primary factor to avoid the risk of crack (primary) appearance during the load application. Recognizing the issue, the specimen length was kept shorter than the predicted mean crack spacing, calculated on the basis of the reinforcement ratio and the bar diameter [20,21]. Furthermore, the current experiment is part of a larger experimental campaign with a broader objective towards the parametric analysis of the bond-slip behavior of RC structures. The bond–slip behavior is affected by a number of parameters, the most important of them being concrete strength, bar diameter, the rib pattern, concrete cover/diameter ratio, and the loading level. The cover/diameter ratio characterizes the confinement conditions. According to the MC2010 [22], concrete is considered well confined when this ratio is not less than the value 5. It has to be higher than 2.5 to prevent splitting failures, although this threshold varies depending on different factors [23,24,25]. In addition to the earlier studies by the authors considering 100 × 100 mm and 150 × 150 mm sections [10,11,17], the current cross-section (200 mm × 200 mm) was chosen to explore the effect of large concrete cover on the bond-slip behavior when good confinement is assured with the cover/diameter ratio being close to 5.

High strength concrete was prepared in the laboratory through the combination of Ordinary Portland Cement (OPC) of grade 42.5R, natural fine aggregate of grade 0/4 mm, coarse aggregate of 5/8 mm grade, and concrete superplasticizer (polycarboxylic ether polymer) to achieve desired workability. It should be noted that the water-cement ratio of the concrete was 0.35. The quantity, specific, and bulk density of the chemical compositions of concrete are mentioned in Table 1.

After 28 days of curing, the mechanical properties of the concrete were evaluated. Standard concrete cylinders (ϕ 150 × h 300), cubes (150 × 150 × 150), and prisms (100 × 100 × 400) were tested to determine the compressive strength, split tensile strength, and flexural strength of concrete respectively. Reinforcing bars of ϕ-20 mm and S500 grade were also tested to find out the yield strength and modulus of the elasticity of steel. Table 2 represents the physical and mechanical properties of the specimen. The average temperature and the relative humidity of the laboratory during the curing period was calculated as 18.1 °C and 94.4%, respectively. It is important to mention that the RC specimen was wrapped with the wet cloths throughout its surfaces for the curing purpose.

**Figure 1:**
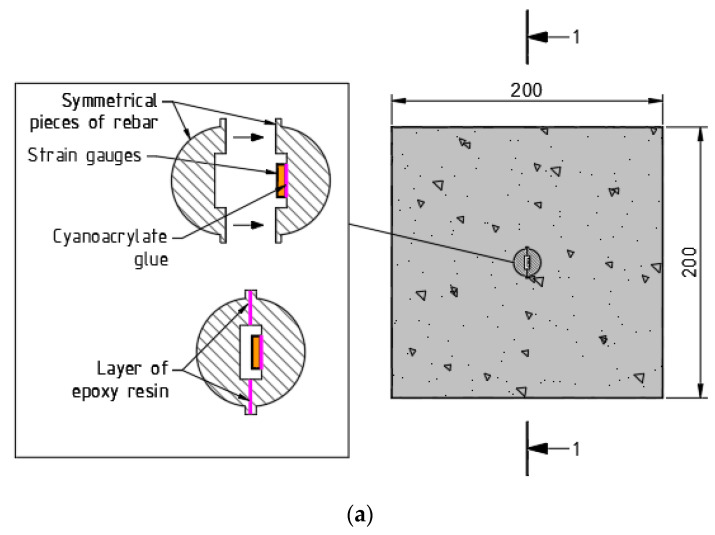

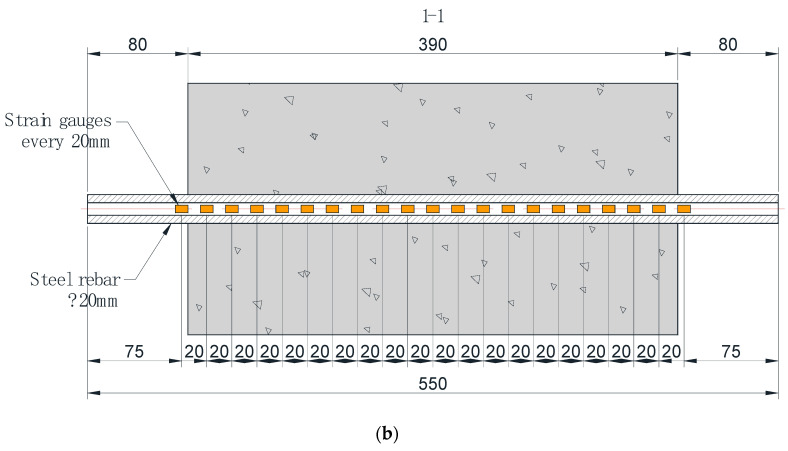
Installation and placement of strain gauge sensors in embedded reinforcement: (**a**) cross-section of the specimen with glued strain gauge sensors inside the modified rebar in detail; (**b**) longitudinal section of the specimen with strain gauge arrangements inside the embedded rebar.

**Figure 2:**
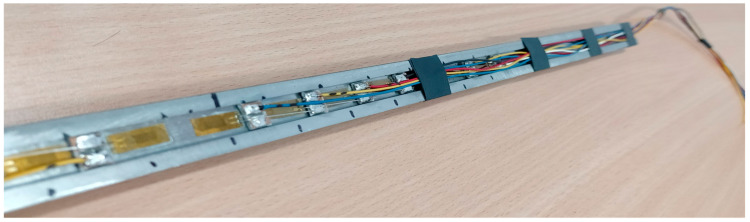
Wiring of strain gauge sensors inside the groove.

Fixing the strain gauge sensors inside the reinforcement bar was undoubtedly the most delicate and time consuming part of this experimental campaign. However, on the positive side, this particular arrangement creates no interruption in the bond performance at the reinforcement-concrete interface, as the devices were placed inside the reinforcement core, not on its surface. At first, a reinforcement bar was cut in half longitudinally, and a groove 2 mm in depth and 10 mm in height was milled on its inner surface, as illustrated in Figure 1a. Two similar halves from two different bars were taken to prepare the new modified bar. The grooves were prepared to accommodate the strain gauge sensors along with associated wirings. In this particular experiment, LY11-6/350 (HBM, Darmstadt, Germany) strain gauges were used, which are linear in type with one measuring grid. Each sensor was about 10 mm wide and contained the nominal resistance of 350 Ω (ohm). In total, 21 sensors were placed with the minimum possible c/c spacing of 20 mm, graphically presented in Figure 1b. For the ease in data analysis, one sensor was placed at the longitudinal center of the reinforcement, followed by an equal number of sensors at both the sides. After gluing the sensors with the cyanoacrylate agent on the longitudinal groove of one half of the bar, they were soldered consciously with required wirings, which were directed out from both the bar terminals. The detailed wiring from the strain gauge sensors inside the reinforcement groove is displayed in Figure 2. In due course, the other half of the bar was adhered with two component epoxy resin glue to prepare the modified full bar. It must be noted that the reduction in the reinforcement area due to the groove was considered in all the calculations. Hereafter, this specimen will be mentioned as SG200×200×390, where the initial part designates the measuring tool strain gauge and the latter part contains dimensions (200 mm×200 mm×390 mm) of the specimen. In addition, similar strain gauge sensors were used outside the specimen for the temperature compensation purpose.

**Figure 3:**
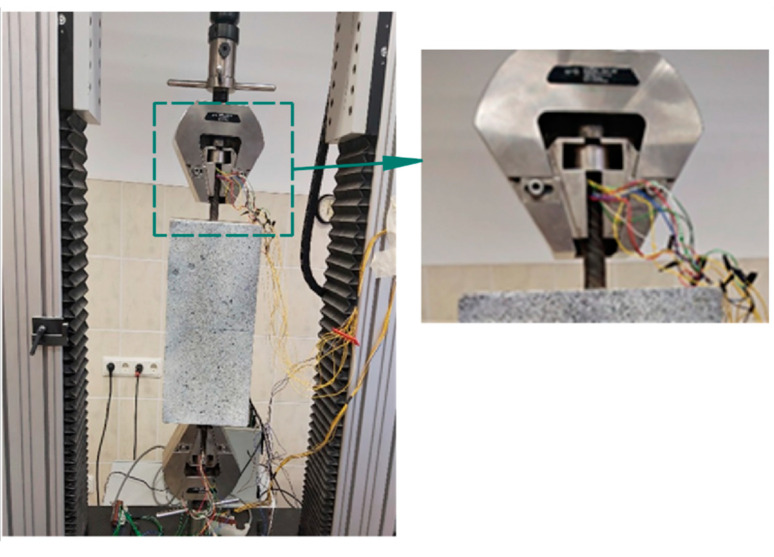
Experimental setup of double pull-out test.

The specimen was tested using a Universal Testing Machine (walter + baiag, Löhningen, Switzerland) with the application of monotonic tensile load in a displacement controlled method. This double pull-out test was well-equipped with LFM100 (walter + baiag, Löhningen, Switzerland), including DION STAT control (walter+baiag, Löhningen, Switzerland) and digital controls along with a data procuring system, as displayed in Figure 3. The loading rate was also maintained at 0.4 mm/min, and was continued until the yielding of the reinforcement. Extra attention was given in the gripping arrangements at the reinforcement terminals to keep the wires undisturbed. The output data from this experiment was collected by the device ALMEMO 5690-2 (AHLBORN, Holzkirchen, Germany) using AMR WinControl (version: 6.9.3.0) Data Acquisition software.

## 3. Finite Element Modelling

FE analysis was frequently performed as a trustworthy tool in the study on reinforcement-concrete interface [26,27], but inadequate in bond-slip analysis [13]. It is found that few researchers dealt with the bond-slip behavior between fiber-reinforced polymer (FRP) reinforcement and concrete by numerically modelled direct pull-out test under tensile [28] and cyclic [29] loading. However, recent trends aiming at the constitutive bond-slip relationship between steel reinforcement and concrete are focusing specifically on the local material properties and bond geometry [30,31]. Thanks to the FE modelling, bond-slip analysis was also numerically performed by providing input parameters of the discrete element method, which further defines the contact element between two materials [32]. In fact, a two-dimensional numerical model was analyzed to investigate the influence of bond-slip on crack spacing [33]. This article makes an attempt to design a three-dimensional rib-scale model of RC prism with identical characteristics of the experimental specimen SG200×200×390. In order to study the reinforcement strain variation with load, a nonlinear FE analysis was performed on the mentioned model, using computer software package ATENA (version: 5.7.0.18967) [34]. From now on, the numerical model will be referred to as FEM200×200×390.

**Figure 4:**
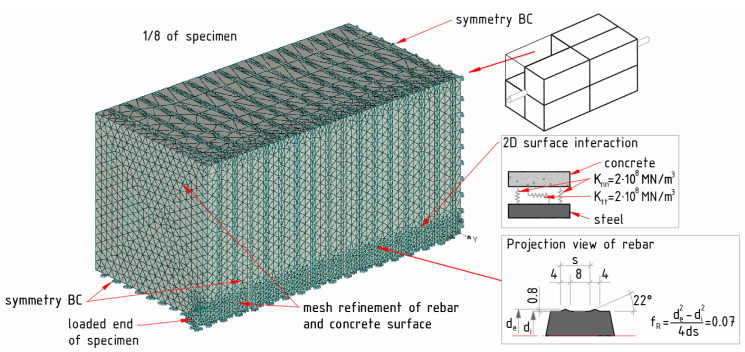
FE Model with important assumptions and boundary conditions.

While designing the model, the authors put special attention on the rib geometry, which has a significant impact on the load transfer mechanism of RC structures [17]. The main assumption of the numerical analysis was the transition of rebar geometry to a finite element model by means of bonding index (*f_R_*), defined as the ratio of the rib surface area’s projection to the surface area between the ribs [35]. For the considered reinforcement bar, the bond index was 0.07. The model features, along with the assumptions, are demonstrated in Figure 4.

For ease and simplicity, 1/8th of the FEM200×200×390 model was considered for further analysis by creating symmetric boundary conditions on relevant surfaces. During the mesh design, the tetrahedral shape of solid isoparametric finite elements with ten nodes were chosen for both the concrete and steel reinforcement. The concrete was modelled as 3D nonlinear cementitious material with the considerations as Young’s modulus $E_{c}=41,526$ MPa, the tensile strength $f_{t}=4.45$ MPa, the compressive strength of cylinder $f_{m,cyl}=71.32$ MPa, and the specific fracture energy $G_{f}=84.3$ N/m [22]. In the case of the mechanical properties of steel, the considerations are as such: yield strength $f_{y}=486$ MPa and modulus of elasticity $E_{s}=201,734$ MPa. The two dimensional interface was implemented between the reinforcement surface and surrounded concrete to mimic the interaction of two materials. The elements at the interface act like springs of appropriate stiffness; essentially, the initial adhesion between two materials. The spring stiffness depends upon the surrounding materials. In our case, the contact layer thickness was assumed to be extremely small. The developed stresses in those springs gradually reached the critical value (predefined by user), which lead to a failure of the interaction surface. Finally, it resulted in a reduced layer, corresponding to friction between two materials [34]. This friction between concrete and reinforcement was evaluated after the interface failure. In this study, the friction coefficient was assumed to be 0.5. It is worth mentioning that the current model sensitivity analysis has shown that quite similar results can be simulated by varying friction coefficient within a range of 0.5–0.6.

The following are the notations of essential parameters of the interaction surface: $f_{t}$ is the threshold value of tensile stresses, c is the cohesion value, φ is the value of dry friction, d  is the nominal diameter of the bar, $d_{e}\;$ and $d_{i}\;$ are the external and internal (core) diameter of the reinforcement bar, respectively, s is the rib spacing, $K_{nn}$ is the initial normal stiffness, and $K_{tt}$ stands for the initial shear stiffness of interaction surface. The latter two variables are recommended to consider based on least possible Young modulus and shear modulus of the surrounding material [34]. In the present study, the normal and shear stiffness of 2 × 10^8^ MN/m^3^ were assumed. It was earlier shown by the authors that, for adequate bond analysis, the interface failure must occur so as to allow the slip between the reinforcement and concrete elements [8]. Consequently, the interface strength, rather than stiffness, becomes the governing parameter controlling the numerical outputs. After the interface failure, the load from reinforcement to concrete is mostly transferred by the direct bearing of the ribs to concrete [8]. Thus, rib height is the second critical parameter for the force transfer from the reinforcement to the surrounded concrete [17]. The rib angle was cautiously chosen as θ=22°, which ultimately produced a good result and made a reasonable agreement with the experimental output. Strain monitoring points were assigned at every 12 mm distance along the reinforcement length.

**Figure 5:**
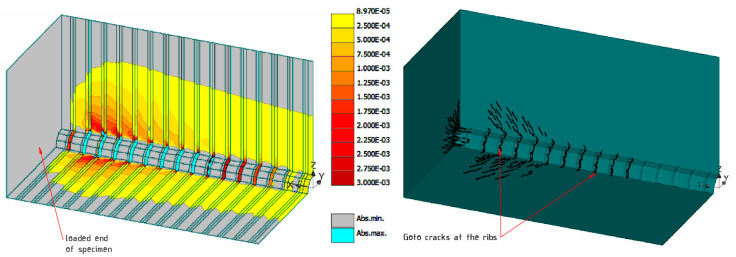
Results of FE analysis: concrete strains and micro-cracking at 110 kN load level.

The load was applied in equally distributed loading steps of 5 kN (1.25 kN for a 1/4th of specimen), until the final step while it was reaching the yield stress of the reinforcement (total 29 steps were analyzed). Results from the FE analysis of the model FEM200×200×390 are illustrated in Figure 5. It displays the concrete strains corresponding to the 110 kN loading level and also the development of small cracks around the rib. These small local cracks radiating out from the deformed bar towards the concrete surface are known as ‘Goto cracks’. They were experimentally shown to separate the inclined concrete studs in a specific pattern around the highly strained longitudinal bars [36]. A threshold of 0.005 mm is chosen to represent crack widths on the current view. For better recognition, strains lower than cracking strains $\varepsilon_{cr}=8.97\cdot 10^{-5}$ are displayed in grey color, and strains higher than 0.003 are presented in cyan color. Extracted strain profiles are discussed in the next chapter of this article.

## 4. Test Results and Analysis

### 4.1. Strain Profile Results

**Figure 6:**
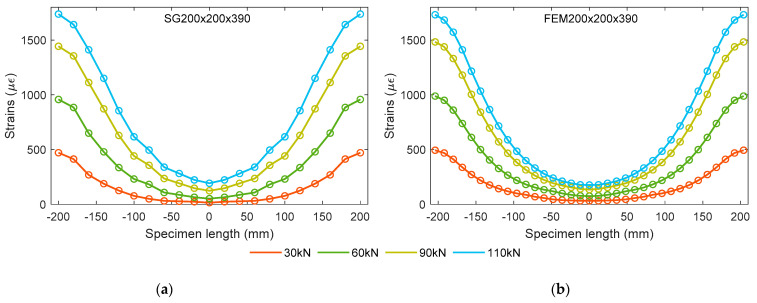
Strain profiles at different load from: (**a**) SG200×200×390 and (**b**) FEM200×200×390.

The authors have demonstrated the laboratory experiment of a double pull-out test and the FE analysis of a numerical model in earlier chapters. The current section presents the strain profiles as the key result from the above mentioned experiments followed by the further analysis. Figure 6 manifests the extracted reinforcement strain results from both experimental specimen SG200×200×390 and numerical model FEM200×200×390. Governed by the yield strength of the reinforcing steel, the theoretical bearing capacity of the prism is about 133 kN. Four different load levels (30, 60, 90, and 110 kN) were chosen to show the variance in strain profiles with the increasing loads. Here, the maximum load value (110 kN) corresponds to the serviceability limit state loading of the specimen. The circular symbols on each strain profile indicate the location of the strain measuring points presented in different intervals in both graphs of Figure 6. This is because in the case of specimen SG200×200×390, the strain gauges were placed 20 mm apart, whereas in numerical model FEM200×200×390, the strain monitoring points were designed at a 12 mm distance along the reinforcement length. It is observed that, in all the curves, the reinforcement strain values are seen at a maximum at the terminals, and gradually decreasing towards the mid-section. This is due to gradually increasing contribution of concrete in force sharing from the terminals towards the mid-section. Based on the mid-section, the extracted strain profiles at different load levels have exhibited a smooth and similar pattern. This indicates the consistency and reliability of the strain measurement technique from the embedded reinforcement based on strain gauges.

It is seen in Figure 6a that the strain curve at the 30 kN load varies from 15 µε to 471 µε, whereas the strain curve at 110 kN load varies from 192 µε to 1737 µε. Evidently, the strain curve gradients are increasing with the increase in load, as seen in both specimens. It can be directly related to the bond stress (τ) as a function of strain gradient through Equation (1).

(1) $$\frac{d\varepsilon_{s}\left(x\right)}{dx}\frac{\emptyset E_{s}}{4}=\tau \left(x\right)$$

where $\varepsilon_{s}$ indicates the reinforcement strain; ∅ is the bar diameter; $E_{s}$ is the modulus of elasticity of reinforcement; and x represents the coordinate of the section considered.

**Figure 7:**
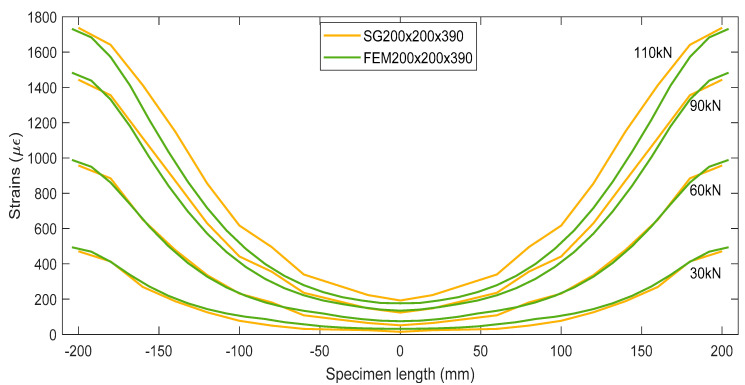
Strain comparison between SG200×200×390 and FEM200×200×390.

Figure 7 directly compares the strain profiles obtained from experimental specimen SG200×200×390 and numerical model FEM200×200×390 at several load levels. At lower load levels such as 30 kN and 60 kN, the strain curves made reasonable agreement with only 7.5% and 8.1% discrepancy, respectively. However, larger errors were noticed at the higher load levels. A maximum 10.2% disparity was observed in strain data at 90 kN and 19.5% at 110 kN. This implies that the numerical model investigated in this study is more reliable at lower load levels, including the service load.

### 4.2. Bond-Slip Behavior

**Figure 8:**
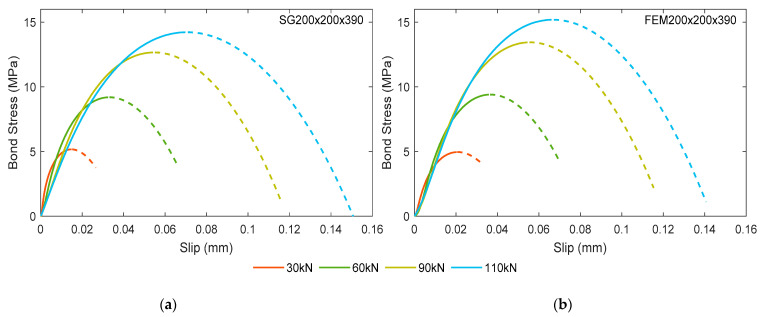
Bond stress-slip behavior at different load levels of: (**a**) SG200×200×390 and (**b**) FEM200×200×390.

In order to determine the bond-slip relation, the obtained strain data were further analyzed based on the stress transfer approach. This method provides the edge to directly model the cracks in accordance with the strain variation within the RC element. It also adopts the theory that the bond stress at the reinforcement-concrete interface directly controls the strains in the reinforcement and concrete through the relative slip [22]. Inherently, it gives a notable advantage in the calculation of bond-slip behavior through this approach. The reinforcement strain profile was assumed to be symmetrical in regard to the mid-section by averaging the strain values of the two halves. The reinforcement strain profile was further numerically smoothed assuming a 4th order polynomial fitting curve. Essentially, bond stress was obtained from the reinforcement strain following Equation (1), and slip was determined as the area between the reinforcement and concrete strain profiles at a given section. For the iterative calculation, the authors thoroughly followed the steps mentioned in a previous literature [10]. As a result, the bond-slip relations of experimental specimen SG200×200×390 and numerical model FEM200×200×390 at four different load levels are calculated and represented in Figure 8.

It is observed from Figure 8 that all the bond-slip curves display a similar parabolic shape until a peak stress; after that, the bond between the reinforcement and concrete is deteriorated. This particular bond-slip format manifests the interactive mechanism between reinforcement and concrete more accurately in comparison with the classical approach [22]. The ascending branches of bond-slip curves at different load levels follow almost the same gradient, confirming that the same bond-slip relationship may be used for the whole loading analysis. A gradual increase in peak bond stress is observed at different load levels for both specimens. For example, Figure 8b displayed the peak bond stresses for FEM200×200×390 as 4.8 MPa, 7.9 MPa, 13.4 MPa, and 15.2 MPa for the loads 30 kN, 60 kN, 90 kN, and 110 kN, respectively. This is due to the increase in strain gradient with respect to the increasing load, clearly visible in Figure 6. It can be considered that the ascending branch of the bond–slip curves may represent the inherent material feature of the reinforcement–concrete interaction, while the descending branch (shown by dashed lines) characterizes the structural effect of cracking on the reduction of bond stresses in the area close to the crack. 

It is important to note that the curves in Figure 8 represent the bond-slip behavior at service load only. The ascending branches of the current test results can be used for developing a law for the ascending branch of a bond-slip law. Indeed, it is an intermediate study that in the future may be used for developing a constitutive law of bond-slip, similar to the ones proposed by Desir et al. [31] or Kanakubo et al. [37]. Similar to the latter research’s findings, earlier findings [8] by the authors explicated the assessment of bond stress reduction at the close proximity of cracks (end of the element) from the descending branch of a bond-slip law.

**Figure 9:**
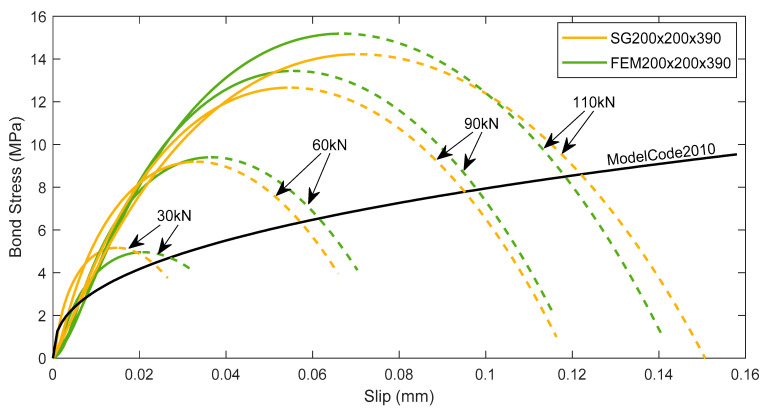
Comparison of bond-slip behavior between SG200×200×390 and FEM200×200×390 at several load levels.

In Figure 9, the ascending branches of the bond-slip relations (depicted by solid lines) representing both the experimental specimen SG200×200×390 and numerical model FEM200×200×390 were compared with the bond-slip model established in MC2010 [22]. Equations (2) and (3) from MC2010 [22] clearly indicate that the bond stress between the reinforcement and concrete is a function of their relative slip. In this case, pull-out failure and good bond condition between the reinforcement and concrete was assumed.

(2) τb=τmax(SS1)α

(3) $$\tau_{max}=2.5\sqrt{f_{ck}}$$

Here, $\tau_{b}$ and $\tau_{max}$ are the bond stress and maximum bond stress respectively, S is the slip, $S_{1}$ and α are taken as 1mm and 0.4 respectively, and $f_{ck}$ is the characteristic compressive strength of concrete in MPa. These equations also limit the slip value as $\left(0\leq S\leq S_{1}\right)$, which is most likely under service conditions, as was the case in the current experiment.

Figure 9 depicts altogether a satisfying correlation between the bond-slip curves from the experimental specimen SG200×200×390 and numerical model FEM200×200×390. Nevertheless, FE analysis slightly overestimated the maximum bond stress by 6.3% and 7% at 90 kN and 110 kN loads, respectively. The largest error at lower loads is quite small: 2.1% at 60 kN, and 3% at 30 kN. It states that the numerical model provides almost accurate predictions of bond-slip behavior, more precisely at the lower load levels. Besides, a clear difference is noticed between the bond-slip relations obtained from the current test and the one suggested by the MC2010 [22]. The code provision distinctly underestimates the peak bond stress comparing to both the experimental and numerical output. For the lowest load level (30 kN), the disparity in peak bond stress is about 16% which is seen to thrive up to 53% for the highest load level (110 kN). The current study clearly demonstrated much stiffer bond response by fast reaching specific values of bond stress at a significantly smaller slip compared to the MC2010 [22]. Such results may be caused by the intrinsic differences in the establishment of bond-slip diagrams. The bond-slip relationship recommended by MC2010 [22] is based on the extensive test results of pull-out specimens, whereas bond-slip diagrams in the present study were derived directly from the measured (or calculated) reinforcement strain distribution. The present study reveals that the bond-slip behavior in tensile RC elements may be significantly different from those observed in the pull-out specimens. Thus, traditional bond-slip relationships may not accurately represent the force transfer mechanism in the tensile zones of RC structures.

## 5. Conclusions

The paper reports the experimental results of the double pull-out tests of a singly reinforced concrete specimen produced from high strength concrete. Reinforcement strains were measured along the reinforcement bar using strain gauges spaced at 20 mm. Alongside a three-dimensional rib-scale, a finite element model was designed and analyzed in ATENA software. Later, its effectiveness was successfully verified with the laboratory test on the basis of the strains and bond-slip results. Subsequently, the bond-slip behavior of the specimens were obtained through stress transfer approach and compared with one prescribed in the MC2010 [22]. Based on the entire experimental-numerical investigation, the following conclusions can be drawn:

- The reinforcement strain profiles recorded at different load levels had a smooth and regular shape, and also kept symmetry in respect to the mid-section of the members representing zero slip. This implies that the employed reinforcement strain measurement technique based on the strain gauge sensors glued inside a groove of the bar may assure consistent and reliable test results.
- The bond-slip relations derived from the experimental reinforcement strain profiles of short RC ties had an ascending and a descending branch. The ascending branches of bond–slip curves were of a parabolic shape, and were in close agreement with each other at different load levels. This confirms that the same bond–slip relationship may be used for the whole loading analysis. Moreover, the ascending bond–slip branch may be considered to represent the inherent material feature of the reinforcement–concrete interaction, whereas the descending branch signifies the structural effect of cracking on the bond stresses in the close proximity of the cracks.
- In the case of strain monitoring, the numerical model performed extremely good correlation with the experimental one at lower load levels, though a maximum 19.5% disparity in strain data was noticed at the highest load level 110 kN.
- In the criteria of predicting bond-slip behavior, the numerical model has shown a thorough consistency in accordance with the experimental result. Only at higher loads has minor disparity (max. 7%) has been noticed.
- The current investigation demonstrated much stiffer bond response by fast reaching specific bond stress values at a significantly smaller slip compared to the MC2010.

The experimental-numerical study of strain monitoring techniques, followed by bond-slip investigation were successful. It creates a vast possibilities for further study related to reinforcement–concrete interaction, such as prediction of cracks, deformation, tension, stiffening, and so on.

## Figures and Tables

**Table 1:** Material Compositions.

Chemical Composition	Quantity (kg/m^3^)	Specific Density (kg/m^3^)	Bulk Density (kg/m^3^)
Ordinary Portland Cement (CEM I 42.5 R)	425	3089	1100
Water-cement ratio 0.35	150	-	-
Fine aggregate 0/4 mm	1165	2650	1620
Crushed coarse aggregate 5/8 mm	715	2610	1310
Concrete plasticizer (1.0%)	4.25	1060 (density of solution)	

**Table 2:** Material Characteristics.

Physical Specification		Mechanical Properties		
Specimen Dimension (mm)	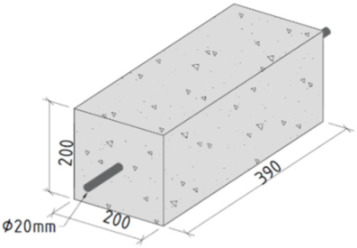	Concrete	*f_m,cyl_* (MPa)	71.32
			*f_m,sp_* (MPa)	4.45
			*f_m,fl_* (MPa)	6.61
			*E_c_* (MPa)	41,526
Groove dimension (mm)	2(2 × 10)	Steel	*f_y_* (MPa)	486
A_S_ (mm^2^)	270.8		*E_s_* (MPa)	201,734

A_S_ = cross-section of the reinforcement, *f_m,cyl_* = compressive strength of concrete, *f_m,sp_* = splitting strength of concrete, *f_m,fl_* = flexural strength of concrete, *E_c_* = modulus od elasticity of concrete, *f_y_* = yield strength of reinforcement, *E_s_* = modulus of elasticity of reinforcement

## Data Availability

The data presented in this study are available on request from the corresponding author.
